# Machine learning methods for automatic pain assessment using facial expression information: Protocol for a systematic review and meta-analysis

**DOI:** 10.1097/MD.0000000000013421

**Published:** 2018-12-10

**Authors:** Dianbo Liu, Dan Cheng, Timothy T. Houle, Lucy Chen, Wei Zhang, Hao Deng

**Affiliations:** a Computer Science and Artificial Intelligence Laboratory, MIT, Cambridge; b Massachusetts General Hospital, Boston, MA; c The First Affiliated Hospital of Zhengzhou University, Henan, PR China.

**Keywords:** accuracy, machine learning, neural networks, pain, prediction

## Abstract

**Introduction:**

Prediction of pain using machine learning algorithms is an emerging field in both computer science and clinical medicine. Several machine algorithms were developed and validated in recent years. However, the majority of studies in this topic was published on bioinformatics or computer science journals instead of medical journals. This tendency and preference led to a gap of knowledge and acknowledgment between computer scientists who invent the algorithm and medical researchers who may use the algorithms in practice. As a consequence, some of these prediction papers did not discuss the clinical utility aspects and were causally reported without following related professional guidelines (e.g., TRIPOD statement). The aim of this protocol is to systematically summarize the current evidences about performance and utility of different machine learning methods used for automatic pain assessments based on human facial expression. In addition, this study is aimed to demonstrate and fill the knowledge gap to promote interdisciplinary collaboration.

**Methods and analysis:**

We will search all English language literature in the following electronic databases: PubMed, Web of Science and IEEE Xplore. A systematic review and meta-analysis summarizing the accuracy, interpretability, generalizability, and computational efficiency of machine learning methods will be conducted. Subgroup analyses by machine learning method types will be conducted.

**Timeline:**

The formal meta-analysis will start on Jan 15, 2019 and expected to finish by April 15, 2019.

**Ethics and dissemination:**

Ethical approval will be exempted or will not be required because the data collected and analyzed in this meta-analysis will not be on an individual level. The results will be disseminated in the form of an official publication in a peer-reviewed journal and/or presentation at relevant conferences.

**Registration:**

PROSPERO CRD42018103059.

## 1 Introduction

Pain is an internal and private experience with complicated neuro-psychosocial mechanisms.^[1]^ Patient's self-report remains to be the golden standard for pain assessment in both medical and computational field,^[2,3–6,7]^ among which Numeric Rating Scale (NRS),^[8]^ and Visual Analog Scale (VAS)^[9–11]^ are the 2 most widely used quantitative pain scales in clinical settings.^[3–6]^ However, these 2 measures are severely subjected to reporting bias due to the nature of self-report,^[12]^ and are influenced by patients’ psychosocial conditions (e.g., catastrophizing,^[13–15]^ and underreporting^[16]^). Another way to measure pain is to measure the intensity based on clinician's observation such as Observer Pain Intensity (OPI) system.^[17,18]^ However, OPI measurement is restricted by human's limited capacity in quantifying pain and heavily relies on the physician's subjective judgment.^[19]^ An objective measure for assessing pain minimizing both reporting bias from patients and observing bias from physicians is needed for research and clinical practice. Quantitative detection of pain in a continuous, automatic and real-time manner will enable timely responses to clinical conditions by physicians and improve hospital experiences of patients.

Despite the fact that humans are capable of reading facial information as a natural facial expression processing system.^[20–22]^ this capacity is limited to simple and large apparent discrepancies in features.^[21,23]^ Naturally, scientists have turned their interests to developing computational algorithms to train machines to decode complicated association between facial expressions and pain.^[3,24]^ Compared with human, machine learning algorithms are able to utilize many different facial features including landmarks, colors, lighting, and movements to detect human emotion. Recent advances in emotion recognition from face image and video benefit significantly from the wide adaptation of convolutional neural networks and increasing volumes of data.^[25]^ Machine-based pain assessment is expected to be more accurate and less biased compared with human observations and its scalability is priceless for clinical utilizations.

### 1.1 Objectives

The primary objective of our meta-analysis is to assess the accuracy (Outcome, O) of automatic machine learning algorithms (Intervention, I) compared with golden standard VAS report (Control, C) for assessing pain intensity among pain patients population (Population, P).^[26]^ In addition, we plan to conduct subgroup analysis to compare accuracy, generalizability, interpretability and computational efficiency by different types of machine learning methods used in order to suggest optimal method for applications in different medical settings. We intend to make suggestions on future strategies of ensemble learning and federated learning, both of which integrate different models, in automatic pain detection. We have conducted a thorough search on PubMed, CoChrane, and PROSPERO databases and our systematic review (SR) and meta-analysis is the first systematic review and meta-analysis on this topic.

## 2 Methods and analysis

### 2.1 Study registration

This protocol review has been registered on PROSPERO (Registration number: CRD42018103059).

### 2.2 Research question development (PICO)

The study research question was developed using the PICO research framework. Details are reported in Table 1.

**Table 1:**
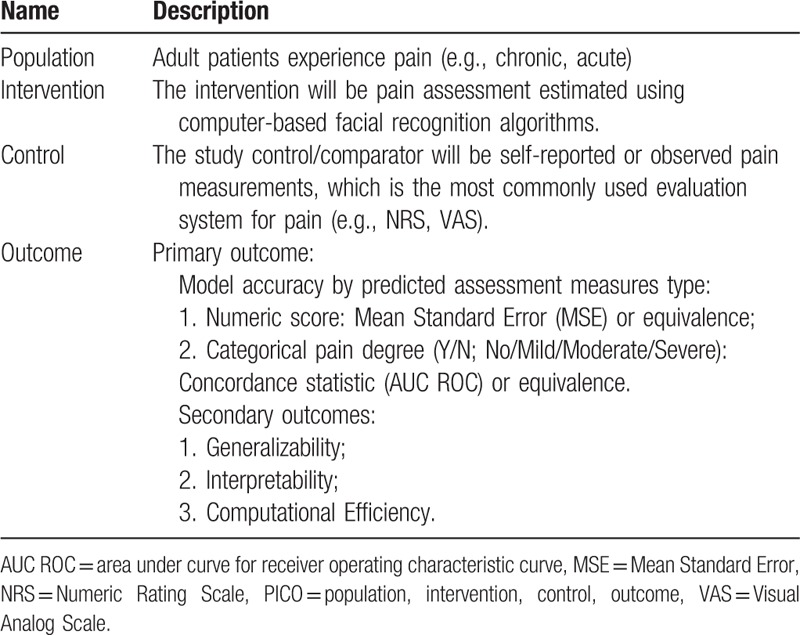
PICO research question development.

### 2.3 Eligibility criteria

All studies in medical settings describing accuracy and performance of machine learning algorithms for automatic pain assessment using human facial expressions are eligible for inclusion. We also include review articles (no related SR and MA articles from our preliminary search) for their reference lists. Exclusion criteria include:

(1) not a human study;
(2) not clinical pain related;
(3) algorithms not based on facial expression information;
(4) not a quantitative study (except reviews);
(5) no measurement of algorithm accuracy (no primary outcome);
(6) clinical pain scores are not used in model building;
(7) facial expression data not in image or video format.

### 2.4 Information source

A global search strategy will be systematically applied in three major public-available electronic medical and technical databases including Web of Science, PubMed, IEEE Xplore Digital Library from 2008 January to most current time (2018 December). Reference lists attached in eligible review articles will be retrieved and screened by author DL and DC. Related professional meeting abstracts and preprints (e.g., IEEE conferences, Pain conferences, arXiv.org) will be searched to account for publication bias. Study language is limited to English.

### 2.5 Searching strategy

Searching strategy is developed using keywords including pain, facial expression, detection, machine learning, deep learning, recognition, and emotion. Details of searching strategy for PubMed and other databases are provided in Table 2.

**Table 2:**
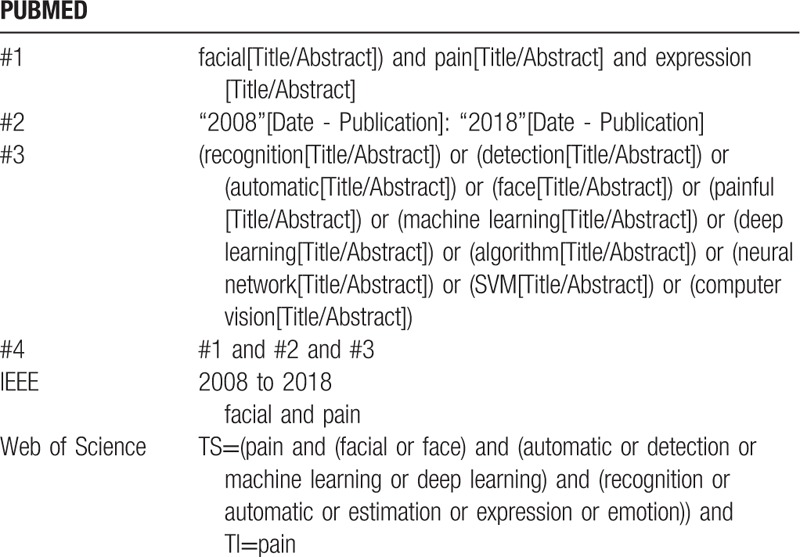
Searching strategy.

### 2.6 Data management

Study record information including title and abstract from searched online databases will be downloaded and imported into Abstrackr platform developed by Brown University.^[27]^ This platform will track and backup all activities when authors conducting the literature review process. Once eligible studies are identified, full-text article will be downloaded data extraction. A data collection sheet is used for study information extraction and storage and this file will be later uploaded to Systematic Review Data Repository (SRDR) website. All data and related logs will be uploaded to Open Science Framework (OSF) website for transparency and version control, if feasible.

### 2.7 Study selection

**Figure 1:**
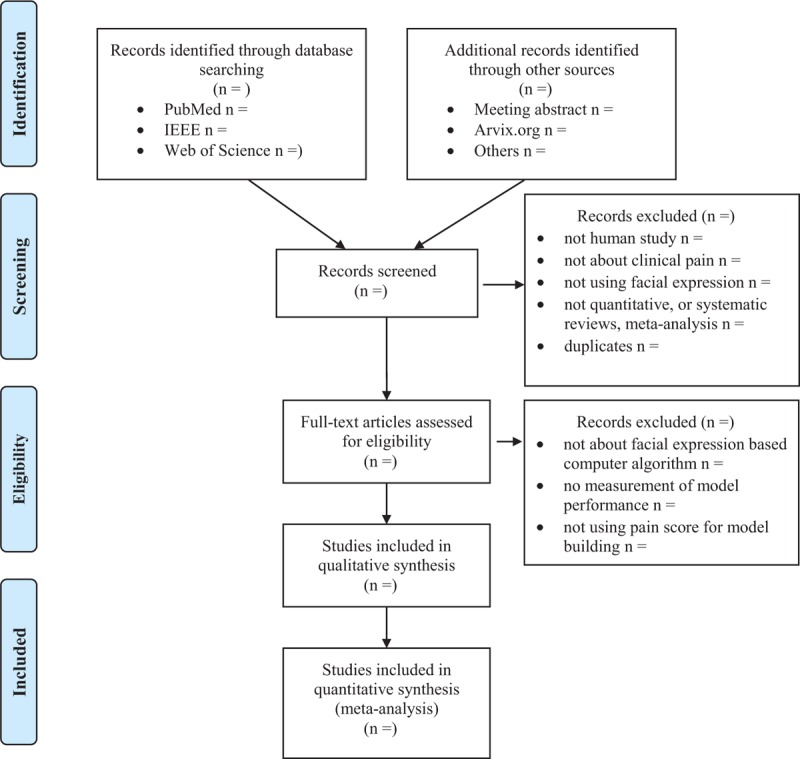
PRISMA 2009 flow diagram.

Two authors (DL and DC) will independently review and screen the titles and abstracts to identify eligible trials according to the inclusion and exclusion criteria using the Abstrackr platform. Disagreements between evaluators were resolved by consensus or consultation with a third investigator (HD or WZ). Excluded studies will be listed in PRISMA flowchart specifying reasons for their exclusion in Figure 1.

### 2.8 Data extraction and collection

The full text will be downloaded and study information will be extracted by DL and DC. They will extract study-level data using a prepared data extraction form. An example of data extraction table is enclosed in Table 3.

**Table 3:**
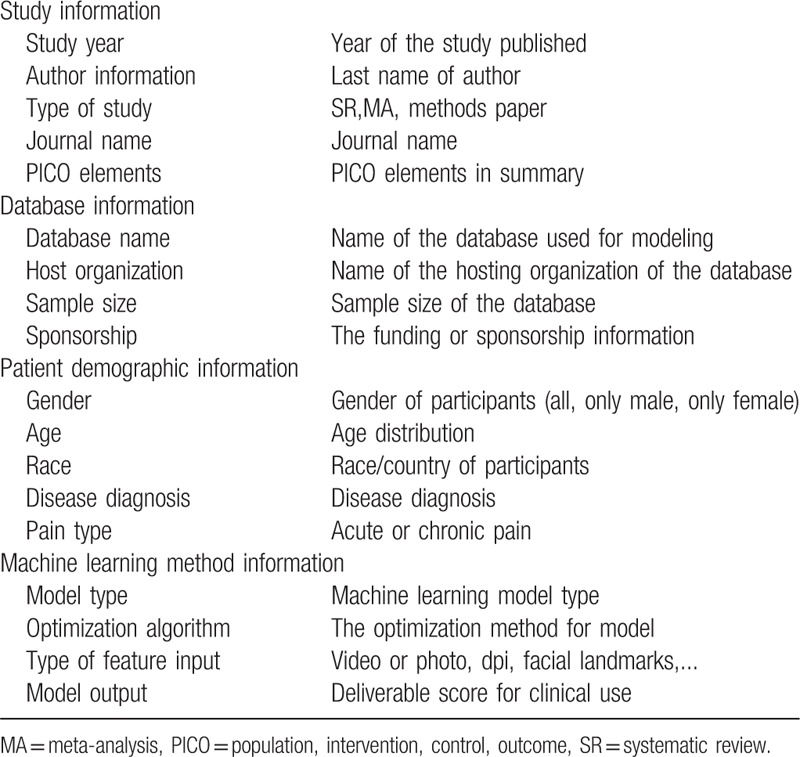
An example of variables collected in data extraction table.

### 2.9 Collected data items

Data items apart from outcomes collected for this meta-analysis will be divided into 4 blocks:

(1) study information including study year, author information, type of study, journal name, and PICO elements;
(2) database information including name of the database used for modeling, name of the hosting organization of the database, sample size of the database, and the funding or sponsorship information;
(3) patient demographic information including gender, age, race, disease diagnosis, and acute or chronic pain;
(4) machine learning method information including machine learning model type, optimization algorithm, and type of input feature.

### 2.10 Machine learning methods

Wide ranges of machine learning methods have been developed for automatic pain prediction from human facial expression. These machine learning methods used in eligible studies can include many different general categories of models like linear regression, Naive Bayes, logistic regression, support vector machine, Gaussian Processes, random forests, genetic algorithms, and artificial neural networks. When analyzing these methods in details, each machine learning model can be represented by several technical attributes including: what features the method utilizes (e.g., facial landmarks, raw face images), the underlying mathematical model (e.g., artificial neural networks, random forests), and the computational algorithm to find the optimum solution (e.g., stochastic gradient descent, Bayesian variational inference^[25,28,29]^). In our study, we will collect information of these attributes mentioned above for each method. An intraclass correlation (ICC) analysis will be applied for subgroup analysis if enough data points are obtained for each category.

### 2.11 Study outcomes

Our outcomes are selected for assessing the overall pain assessment performance of studied machine learning method. The primary outcome is model accuracy estimate (e.g., area under curve for receiver operating characteristic curve [AUC ROC]; F1 score, and proper score function such as brier score if available) to correctly predict pain intensity. Secondary outcomes include different aspects of utility measures such as generalizability, interpretability, and computational efficiency.

#### 2.11.1 Primary outcome: standardized measurement of model predicting accuracy

We expect the high degree of heterogeneity in experimental setting, populations, methods, outcome reporting, therefore, the primary goal is a descriptive summary of these issues. Predicting accuracy of the model typically shall include 2 parts of information: accuracy and calibration. However, computer science studies rarely report calibration results; therefore, our study will mainly focus on the accuracy performance of predictive accuracy of machine learning models. For regression algorithms, all measurements of error measurement, including Mean Absolute Error (MAE), will be converted to Mean Square Error (MSE) for comparison if possible. All correlation measurements will be converted to ranked correlation (Spearman correlation). For classification algorithms, all the accuracy measurement will be converted to AUC ROC and F1 score. The measurements that cannot be standardized will be reported as original values. If diagnostic test accuracy (DTA) measures including sensitivity and specificity were reported, this information will also be collected and analyzed depending on study data availability.

#### 2.11.2 Secondary outcomes: generalizability, interpretability and computational efficiency

For descriptive purpose only, a subjective comprehensive judgment will be given to each method at the model level about how generalizable and interpretable the model is. The levels of the judgment rank from High, Moderate, Low and Very Low. Computational efficiency will be analyzed if benchmark time for running the model is provided.

### 2.12 Incomplete information and missing data

If essential information is missing, we will attempt to collect the data by contacting the authors of the studies. If we fail to obtain sufficient data, these studies will be omitted from the data synthesis.

### 2.13 Risk of bias in individual studies

A novel risk of bias evaluation tool will be custom designed for this study similar to the Cochrane Risk of Bias tool.^[30]^ The risk of bias in eligible studies will be evaluated at 3 domains including:

(1) input data selection,
(2) model performance and
(3) result reporting.

Factors influencing input data selection include database sponsorship (e.g., organization or single study data), and image/video quality (e.g., Dpi of video, camera setting); Factors influencing model performance include research team (e.g., whether there is a professional computer scientist or mathematician), innate prior of machine learning algorithm, algorithm training process, and evaluation method. Factors introducing reporting bias include incomplete reporting, selective reporting, non-standard reporting (e.g., only report point estimate without standard errors or confidence intervals). Based on these domains, risk of bias of eligible studies will be categorized into low risk, moderate risk, high risk, and unclear and presented. In as separate effort to demonstrate the quality of included pain prediction studies, our group plans to compare reported items in eligible studies with the recommended reported items according to Transparent Reporting of a multivariable prediction model for Individual Prognosis Or Diagnosis (TRIPOD) statement.^[31]^

### 2.14 Statistical analysis and data synthesis

As different model, features and gold standard are used in different studies, we plan to synthesize model accuracy performances taking both model calibration and accuracy into account. Hosmer-Lemeshow chi-square test and ranked correlation will be used for assessing calibration on classification and regression models, if applicable. As described in the previous section, the C-statistic (AUC ROC) for classification model and MSE for regression model along with their 95% confidence intervals will be used for assessing accuracy. The Galbraith plot, Higgins and Thompson I-square will be used to assess heterogeneity among the studies. If no evidence of statistical heterogeneity is detected, we will use a fixed-effects model. If considerable heterogeneity is indicated (I-square >50%), we will pool the summary measures across the studies using random-effects model optimized using Laird and DerSimonian method. Additionally, we will also search for the possible sources of heterogeneity from both clinical and methodological perspectives to provide an explanation or will consider conducting subgroup analysis. Meta-regression will be considered, if applicable. Extracted outcome data stored in SRDR will be imported into RevMan V.5.2.1 software and R V3.3.2 for analyses.

### 2.15 Subgroup analyses

We intend to conduct subgroup analyses by machine learning model types (e.g., regression vs classification; neural networks vs traditional machine learning), facial data input format and pain condition (e.g., chronic pain vs acute pain), if feasible.

### 2.16 Publication bias

We will search related professional meeting abstracts and technical preprints to account for publication bias. Publication bias will also be assessed using Contour-Enhanced Funnel Plots.

### 2.17 Confidence in cumulative evidence

Confidence in cumulative evidence will be conducted in accordance with the GRADE guideline. Inconsistency will be assessed using I-square test and Galbraith plot as described in the previous section. Indirectness will be assessed by examining the collected PICO elements of eligible studies and comparing generalizability (one of our second outcomes). Imprecision will be assessed by examining the study sample sizes and confidence intervals of interesting outcomes.

## 3 Discussion

In the era of artificial intelligence, computers start to outperform human in many fields. Machines now can perform well in identifying movements and certain behaviors from image and video information. These technical advancements provide a potential opportunity for automatizing pain detection and assessment using machine-observed facial information in real-world clinical settings. However, curation of large interventional study data sets of human pain scores with facial expression information is still challenging with both practical difficulties and ethical concerns. This lack of training data limits both accuracy and generalization of trained machine learning models. Additionally, good interpretability and computational efficiency are important elements for real-time information streaming between patients and clinicians for clinical utility. In this study, we propose a protocol for a systematic review and a meta-analysis on machine learning methods in automatic pain assessment from facial expression aiming to provide a useful reference for implementation of automatic pain management and collection of patient-produced data for clinicians and researchers.

## Author contributions

**Conceptualization:** Dianbo Liu, Dan Cheng, Wei Zhang, Hao Deng.

**Data curation:** Dianbo Liu, Dan Cheng, Hao Deng.

**Formal analysis:** Dianbo Liu, Dan Cheng.

**Funding acquisition:** Wei Zhang.

**Methodology:** Dianbo Liu, Dan Cheng, Timothy Houle, Lucy Chen, Wei Zhang, Hao Deng.

**Project administration:** Wei Zhang, Hao Deng.

**Resources:** Wei Zhang, Hao Deng.

**Supervision:** Wei Zhang, Hao Deng.

**Validation:** Dianbo Liu, Dan Cheng.

**Visualization:** Dianbo Liu, Dan Cheng.

**Writing – original draft:** Dianbo Liu, Dan Cheng, Hao Deng.

**Writing – review & editing:** Timothy Houle, Lucy Chen, Wei Zhang, Hao Deng.
